# Exposure to green space is associated with higher skin microbiota species richness in children

**DOI:** 10.1093/pnasnexus/pgaf115

**Published:** 2025-05-19

**Authors:** Thessa Van Pee, Hanne Croons, Esmée Bijnens, Doris Vandeputte, Eleni Renaers, Hanne Sleurs, Lore Verheyen, Nick Giesberts, Maartje Vangeneugden, Leen Rasking, Michelle Plusquin, Janneke Hogervorst, Tim S Nawrot

**Affiliations:** Centre for Environmental Sciences, Hasselt University, Agoralaan Building D, 3590 Diepenbeek, Belgium; Centre for Environmental Sciences, Hasselt University, Agoralaan Building D, 3590 Diepenbeek, Belgium; Centre for Environmental Sciences, Hasselt University, Agoralaan Building D, 3590 Diepenbeek, Belgium; Department of Environmental Sciences, Open Universiteit, Valkenburgerweg 177, Heerlen 6419, The Netherlands; Lab of Microbiology LM-UGent, Department of Biochemistry and Microbiology (WE10), Ghent University, K.L. Ledeganckstraat 35, Ghent 9000, Belgium; Centre for Environmental Sciences, Hasselt University, Agoralaan Building D, 3590 Diepenbeek, Belgium; Centre for Environmental Sciences, Hasselt University, Agoralaan Building D, 3590 Diepenbeek, Belgium; Centre for Environmental Sciences, Hasselt University, Agoralaan Building D, 3590 Diepenbeek, Belgium; Centre for Environmental Sciences, Hasselt University, Agoralaan Building D, 3590 Diepenbeek, Belgium; Centre for Environmental Sciences, Hasselt University, Agoralaan Building D, 3590 Diepenbeek, Belgium; Centre for Environmental Sciences, Hasselt University, Agoralaan Building D, 3590 Diepenbeek, Belgium; Centre for Environmental Sciences, Hasselt University, Agoralaan Building D, 3590 Diepenbeek, Belgium; Centre for Environmental Sciences, Hasselt University, Agoralaan Building D, 3590 Diepenbeek, Belgium; Centre for Environmental Sciences, Hasselt University, Agoralaan Building D, 3590 Diepenbeek, Belgium; Department of Public Health and Primary Care, Leuven University, Herestraat 49-box 706, 3000 Leuven, Belgium

## Abstract

Skin is the exterior interface of the human body with the environment and harbors millions of microorganisms crucial for skin health. Associations between early-life green space exposure and the skin microbiome of children remain unstudied. Skin swabs were collected from 402 children (4–12 years old) enrolled in the ENVIR*ON*AGE birth cohort. Skin alpha diversity indices and the relative abundance at family and species levels were determined using 16S rRNA gene HiFi amplicon sequencing. Total green, high-growing green, and low-growing green were estimated in several radii around their current residential and school address based on high-resolution land cover data. Multiple linear regression models between green-space indices and skin microbiome alpha diversity indices were adjusted for sex, age, frequency of soap use, maternal education, season of skin swab collection, sequencing batch, and storage duration of the skin swab. As interaction terms between green-space indices and season were borderline statistically significant, we also ran the linear regression models stratified by season. Last, we performed a differential relative abundance analysis, accounting for the covariables above. Total green and high-growing green in multiple radii (from 100 to 500 m) were positively associated with observed richness (regression coefficients ranging from 10.06 to 15.31 [*P*-value ranging from 0.03 to 0.12] per interquartile range increase in green). The associations were only statistically significant when skin swabs were collected in the warm season. The relative abundance of the bacterial families *Xanthomonadaceae*, *Intrasporangiaceae*, *Pseudomonadaceae*, and *Caulobacteraceae* was statistically significantly positively associated with total and high-growing green within 300 m. Our findings suggest an influential role of early-life green space exposure on skin microbiome composition. Additional research is needed to investigate whether the observed positive relationship between green space and skin bacterial richness has implications for human health.

**Significance Statement**

The skin microbiome is vital for skin health, aiding in skin maturation, immune modulation, and wound repair. While adult studies showed environmental influences (e.g. green space) on skin microbial composition, child-focused research remains limited. Our findings show that exposure to total green and high-growing green is positively associated with skin microbiota species richness and the relative abundance of different bacterial families in children aged 4 to 12 years. We found these results, especially in small radii (100 to 500 m) and during summer. Further research is needed to determine whether the observed positive association between green space and skin bacterial richness has implications for human (skin) health, as this would highlight the importance of ensuring adequate green space in residential and school environments further.

## Introduction

The skin is the exterior interface of the human body with the environment, making it vulnerable to external factors (1, 2). Despite its dry, nutrient-deficient, aerobic, and acidic habitat, it harbors millions of microorganisms (2), essential for skin health. For instance, skin microbes produce protease enzymes that aid in the renewal and maturation of the stratum corneum (1, 3). Additionally, they can activate T cells, contributing significantly to wound repair and closure (4). Furthermore, they provide defense against pathogen invasion by producing, e.g. antimicrobial peptides (5). Several studies observed associations between changes in the commensal skin microbiome and dermatological disorders, including psoriasis (6) and acne (7), and even systemic diseases, such as cardiovascular disorders (8). Skin microbiome composition is influenced by intrinsic (e.g. ethnicity and age) and extrinsic factors (e.g. mode of delivery, antibiotics, and hygiene levels [e.g. use of water and soap]) (1), but remains relatively stable over time (9).

In the framework of human health, there has been growing attention to the importance of green space, as positive associations with green space and physical activity (10), reduced stress (11), and cardiovascular health (12) have been observed. Research has also focused on the relationship between the surrounding environment and the skin microbiome. For instance, in a study by Selway et al. (13), skin microbial richness and phylogenetic diversity increased, and microbial skin composition resembled the soil microbiome more closely when adults mimicked the behavior of children interacting with the environment (i.e. digging in dirt and brushing against vegetation). Furthermore, a study on dogs showed that lifestyle and residential environment independently influence the skin microbiome composition (14). The findings indicated that a rural lifestyle and environment were linked to a higher abundance of environmental bacteria, whereas an urban lifestyle and living environment were associated with a greater presence of taxa common in built environments, such as *Chroococcidiopsis*. Green space exposure may influence the skin microbiome via multiple pathways, including mitigating harm (e.g. reducing air pollution) or increasing social encounters (15, 16). Yet, the most plausible pathway involves the transfer of environmental microbes (17). This transfer can occur directly when humans come into contact with trees and grasses, but it can also happen indirectly when emitted pollen, which carries microbes, comes into contact with the skin. Lastly, when grass is mowed or leaves fall from trees, microbes can become airborne and adhere to the skin (18). This human–environment transfer of microorganisms is bidirectional, as human commensal and pathogenic bacteria have been found on plant leaves of outdoor plants (19).

To date, the relationship between early-life exposure to green space and the skin microbiome of children remains unstudied. To our knowledge, we present the first study in which the association between green space exposure and skin microbiome alpha diversity is cross-sectionally investigated in children. This study combined the percentage of green space at the child's residential and school address for 402 children enrolled in the ENVIRonmental influence ON early AGEing (ENVIR*ON*AGE) birth cohort (20). In addition to assessing alpha diversity (richness, evenness, and diversity), we computed the relative abundances at family and species levels to examine which specific bacterial taxa are associated with exposure to green space.

## Materials and methods

### Study population and sample collection

The ongoing prospective ENVIR*ON*AGE birth cohort recruits mother–newborn pairs at arrival for delivery in the East-Limburg Hospital (ZOL; Genk, Belgium) and follows them longitudinally (20). The cohort is approved by the Ethical Committees of Hasselt University and East-Limburg Hospital (EudraCT B37120107805) and complies with the Declaration of Helsinki. At delivery, detailed sociodemographic information about the mother–child pair is collected via questionnaires (e.g. ethnicity, parity, and maternal education) and medical records (e.g. newborn sex and day of delivery). Ethnicity is classified as “European” when two or more grandparents are of European descent and as “non-European” when at least three grandparents are of non-European origin. Parity is categorized as first, second, or third or more children. Maternal education is coded “low” when the mother did not obtain a high school diploma, “middle” when the mother obtained a high school diploma, and “high” when the mother obtained a college or university degree. After 4 to 6 years and 9 to 11 years, mother–child pairs are contacted again to participate in the follow-up phases in which, among other samples and measurements, skin bacteria are sampled by wetting a FLOQSwab (COPAN Diagnostics) with a drop of sterile saline and rubbing the forehead of the child for 1 min. Swabs are immediately stored in 1 mL eNAT buffer at −20 °C to inactivate microbial viability and stabilize bacterial DNA. During the follow-up phases, questionnaires are administered regarding lifestyle (e.g. use of soap while washing the body, residential presence of furry pets, school address, and time spent outdoors) and medical history (e.g. skin disorders and prior antibiotic use). Written informed consent is obtained from the participants at birth and the follow-up phases. A total of 403 skin swabs were collected from children participating in the follow-up studies between the 2021 May 12 and the 2023 June 31 and were used in the present cross-sectional analyses (Fig. S1).

### Skin microbiome 16S rRNA gene sequencing and processing

DNA from 403 samples and six negative controls (i.e. two Eppendorf tubes with sterile saline solution, two Eppendorf tubes with eNat buffer, and two Eppendorf tubes with Enat buffer and FLOQSwab) was extracted using the DNeasy PowerSoil Pro Kit (Qiagen). We followed the manufacturer's protocol with minor adaptations to enhance the DNA yield: before the extraction, skin swab eNAT buffers were centrifuged at 5,000 rpm for 10 min and the top 500 µL was discarded, and 800 µL CD1 was replaced by 600 µL CD1 and 200 µL of phenol-chloroform-isoamyl alcohol (25:24:1). The extracted DNA was eluted in 25 µL 10 mM Tris and stored at −80 °C. Further sample processing and analyses were performed by VIB Nucleomics Core (www.nucleomics.be). In brief, DNA concentration and purity were determined spectrophotometrically using the Nanodrop ND-8000 (Nanodrop Technologies) and Qubit Flex fluorometer (Invitrogen). An equal amount of DNA template was used for each sample to amplify the 16S rRNA gene (∼2 ng total DNA per reaction or a fixed volume if quantification failed due to low DNA quantity). PCR conditions were taken from the PacBio protocol (PN 101-599-700 REV05 MAY2022). A negative control (buffer) and a positive control sample (ZymoBIOMICS Microbial Community DNA Standard: D6305) were included with separate barcode combinations as technical controls. Amplicons were converted into SMRTbells following the PacBio guidelines and using the SMRTbell kit 3.0 (PN 102-359-000 REV 02 SEP2022). The SMRTbells were complexed with polymerase and loaded on the Sequel IIe device for 16S rRNA gene HiFi amplicon sequencing. Data were received as HiFi FASTQ files and processed via the PacBio full-length 16S nextflow pipeline v0.6. Sequences were demultiplexed and subsequently underwent quality control and primer removal using DADA2. Only reads with a QV value >20 were included, the full length of the F27-R1492 16S primers was trimmed at both ends of each read, MaxEE was set to 2, and maxN to 0. Only reads with a length between 1,000 and 1,600 base pairs were included in the analysis. Chimeras were removed using the removeBimeraDenovo() function in Rstudio and an amplicon sequence variant (ASV) table was generated based on the remaining reads. Taxonomy was assigned using the VSEARCH and Naïve-Bayes classifier Flexible VSEARCH database based on 97% overlap. The resulting ASVs (in total of 90,553 ASVs), and taxonomy tables were combined with the metadata file into a phyloseq object (Phyloseq, version 1.26.1) (21). Contaminant ASVs were removed using the combined frequency (threshold is 0.1) and prevalence (threshold is 0.5) method from the R package Decontam (version 1.2.1) (22). A total of 1,452 ASVs were removed. Next, ASVs that were present in less than three samples and that had less than five reads in total were omitted from the dataset. The number of remaining ASVs was 8,830. Relative taxa abundances were computed at family and species levels for samples with >500 reads (*n* = 402). To calculate bacterial alpha diversity indices (observed richness, species evenness, and Shannon diversity), samples were rarefied to 3,000 reads (*n* = 380). The flow of sample and ASV selection is depicted in Fig. S1.

### Green space

Exposure to green space was assessed, as previously described (23). In brief, the residential and school addresses of the child at the time of the skin swab collection were geocoded using Geographic Information System (GIS) functions with ArcGIS 10 software (Esri Inc.). Green space was estimated in several radii (i.e. 100, 300, 500, 1,000, and 2,000 m) around the residence and school using the Green Map of Flanders 2012, which is a high-resolution (1 m (2)) land cover dataset from the Agency for Geographic Information Flanders. Three different green-space variables were assessed based on the map: high-growing green (vegetation with a height >3 m), low-growing green (vegetation with a height <3 m), and total vegetation cover (i.e. total green; the sum of the former two). Exposure to green space at the residence and school was combined based on the child's calculated average proportion of time spent at school.

### Statistical analyses

All statistical analyses were performed using Rstudio (version 4.2.3; R Core Team). First, linear regression models were used to associate skin microbiome indices (i.e. observed richness, Shannon diversity, and species evenness) with green space. We incorporated different green-space metrics (total green, high-growing green, and low-growing green) in different radii (100 to 2,000 m) to identify which type of green space may be essential and whether a direct proximity to green space may have a greater impact than more distant green space. In these multiple linear regression models, we accounted for a priori selected covariables: the child's sex (male or female), age (in years), frequency of soap use (never, 1–2 times/week, 3–4 times/week, 5–6 times/week, 7 or more times/week), maternal education (low, middle, or high), season in which the skin swab was collected (winter, spring, summer, or autumn; based on astronomical seasons), sequencing batch (first, second, or third), and storage duration of the skin swab (in days). Q–Q plots of the residuals were inspected to check the assumptions of linear models. For these analyses, the skin swab microbiome data of 380 children were used (see above and Fig. S1). The results were expressed as a difference in skin microbiome index (with 95% CI) per interquartile range (IQR) increase in green space exposure. Reported *P*-values were two-tailed and considered statistically significant when ≤0.05. In sensitivity analyses, we assessed whether adjusting for the residential presence of furry pets (yes or no), ethnicity (European or non-European), short-term air pollution exposure (prior week PM_2.5_), long-term air pollution exposure (prior year PM_2.5_), passive smoke exposure (currently, previously, or no), indoor mold (yes or no), parity (first, second, or third or more children), skin disorders (yes or no), previous month or year antibiotic use (yes or no), and time spent outdoors (times/week), or whether only considering green space at the residential address, or only including children who had lived for at least 1 year at the current residential address (*n* = 366), or only children born via a natural delivery (*n* = 368) affected the observed associations. Air pollution exposure was calculated through high-resolution spatial-temporal interpolation models (20). The season in which the skin swab was collected is related to how green the green space is and may influence green space exposure potentially in other important ways. Therefore, we ran the multiple linear regression models with an interaction term on a multiplicative scale between green space and season (warm [summer and spring combined] versus cold [autumn and winter combined]) and stratified the analyses by season (warm versus cold) to examine whether findings were season specific. Lastly, raw family and species counts were used to perform a differential relative abundance analysis (*n* = 402) using the “Analysis of Compositions of Microbiomes with Bias Correction (ANCOM-BC2)” R package (version 2.0.2), while accounting for the covariables above. All options remained as default. Only bacterial families and species present in ≥10% of all participants were included in the analysis. Multiple testing was corrected by setting the two-tailed false-discovery rate (FDR) *q*-value at ≤0.10. Results were expressed as a percentage difference in the relative abundance per IQR increase in green space exposure.

## Results

### Population characteristics

Our study comprised of 402 children, of which 185 (46%) were boys with a mean ± SD age of 8 ± 3 years. The majority of the children were the first born (54%) via a natural delivery (96%) and were of European descent (96%). Most of the children used soap 1–2 times/week (38%) or 3–4 times/week (38%) while washing, did not have a skin disorder (85%), and did not take antibiotics in the month (95%) or year (77%) prior to the skin swab collection. Approximately, half of the households (48%) had a furry pet. The vast majority of children were not exposed to passive smoke (85%) or indoor mold (96%) and played on average 6 ± 4 times/week outdoors. Most mothers had a higher education degree (74%). Skin swabs were mainly collected in summer (34%), followed by winter (23%), spring (23%), and autumn (21%; Table 1). Skin swabs were analyzed after, on average, 109 ± 100 days and sequenced in three more or less equal batches (batch 1: 29%, batch 2: 38%, batch 3: 33%). Table 2 shows the distribution of green-space variables in several radii around the residential and school addresses.

**Table 1.** Study population characteristics.

Characteristics	Participants (*n* = 402)
*Child*	
Sex	
Boy	184 (45.77%)
Girl	218 (54.23%)
Age (years)	7.85 ± 2.59
Ethnicity	
European	386 (96.02%)
Non-European	16 (3.98%)
Use of soap while washing (times/week)	
Never	16 (3.98%)
1 or 2	155 (38.56%)
3 or 4	153 (38.06%)
5 or 6	41 (10.20%)
7 or more	37 (9.20%)
Skin disorder	
Yes	47 (11.69%)
Do not know	13 (3.23%)
No	342 (85.08%)
Previous month antibiotic use	
Yes	19 (4.73%)
No	383 (95.27%)
Previous year antibiotic use	
Yes	93 (23.13%)
No	309 (76.87%)
Time spent outdoors (times/week)	6.05 ± 3.45
Passive smoke exposure^a^	
Currently exposed	44 (11.46%)
Previously exposed	13 (3.39%)
Not exposed	327 (85.16%)
Furry pet	
Yes	191 (47.51%)
No	211 (52.49%)
Indoor mold^a^	
Yes	17 (4.43%)
No	367 (95.57%)
*Maternal*	
Parity	
First child	217 (53.98%)
Second child	142 (35.32%)
Third or more children	43 (10.70%)
Partus	
Natural delivery	386 (96.02%)
Cesarean section	16 (3.98%)
Maternal education	
Low	7 (1.74%)
Middle	98 (24.39%)
High	297 (73.87%)
*Skin microbiome*	
Season of skin swab	
Winter	91 (22.64%)
Spring	91 (22.64%)
Summer	135 (33.58%)
Autumn	85 (21.14%)

Data are presented as mean ± SD or total number (percentage). Ethnicity was based on the native country of the newborn's grandparents and described as European when two or more grandparents were European or non-European when at least three grandparents were of non-European origin. Maternal educational level was coded “low” if the participant did not obtain a high school diploma, “middle” if the participant obtained a high school diploma, and “high” if the participant obtained a college or university degree.

^a^Data available for 384 participants.

**Table 2.** Descriptive statistics for green space exposure variables and skin microbiome alpha diversity indices.

	25th percentile	Mean	75th percentile
*Green-space residence-school combined*			
Total green			
100 m (%)	40.40	48.81	58.02
300 m (%)	42.89	51.20	60.04
500 m (%)	41.46	51.50	62.09
1,000 m (%)	41.21	52.42	65.14
2,000 m (%)	29.65	42.05	53.27
High-growing green			
100 m (%)	7.61	16.17	21.38
300 m (%)	11.37	20.91	28.02
500 m (%)	12.85	23.36	31.30
1,000 m (%)	16.90	28.31	40.15
2,000 m (%)	20.32	32.97	44.24
Low-growing green			
100 m (%)	26.36	32.64	39.11
300 m (%)	25.12	30.28	36.15
500 m (%)	22.67	28.14	32.97
1,000 m (%)	19.71	24.11	27.81
2,000 m (%)	7.19	9.07	10.72
Skin microbiome alpha diversity			
Observed richness	225.25	300.51	373.75
Shannon diversity	4.13	4.46	4.95
Species evenness	1.73	1.81	1.95

Green space exposure values are expressed as percentages.

### Sequencing data and bacterial relative abundance of skin microbiome

**Fig. 1.**
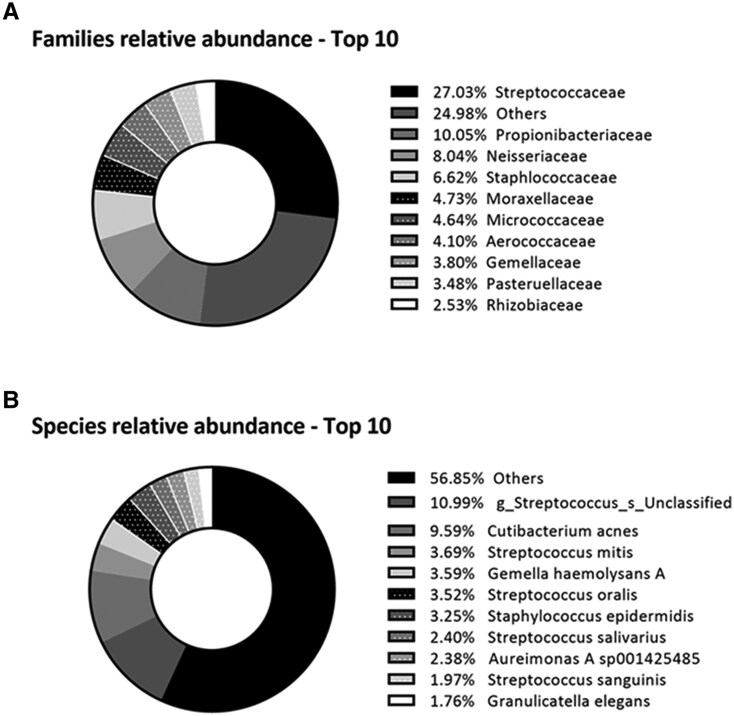
Overview of the relative abundance (%) of the 10 most abundant bacterial A) families and B) species in relation to all other taxa.

A total of 402 skin swabs, with an average ± SD of 10,800 ± 6,559 reads and 379 ± 169 ASVs postfiltering, were included in the analyses. Bacterial relative abundances were calculated at the family (182 families; Fig. 1A) and species level (1,191 species; Fig. 1B). The most abundant families were Streptococcaceae (27%), Propionibacteriaceae (10%), and Neisseriaceae (8%); the most abundant species were an unclassified species belonging to the genus *Streptococcus* (11%), *Cutibacterium acnes* (10%), and *Streptococcus mitis* (4%). Next, skin microbiome alpha diversity indices were calculated using the ASV table (Table 2).

### Associations between green space exposure and skin microbiome alpha diversity

**Fig. 2.**
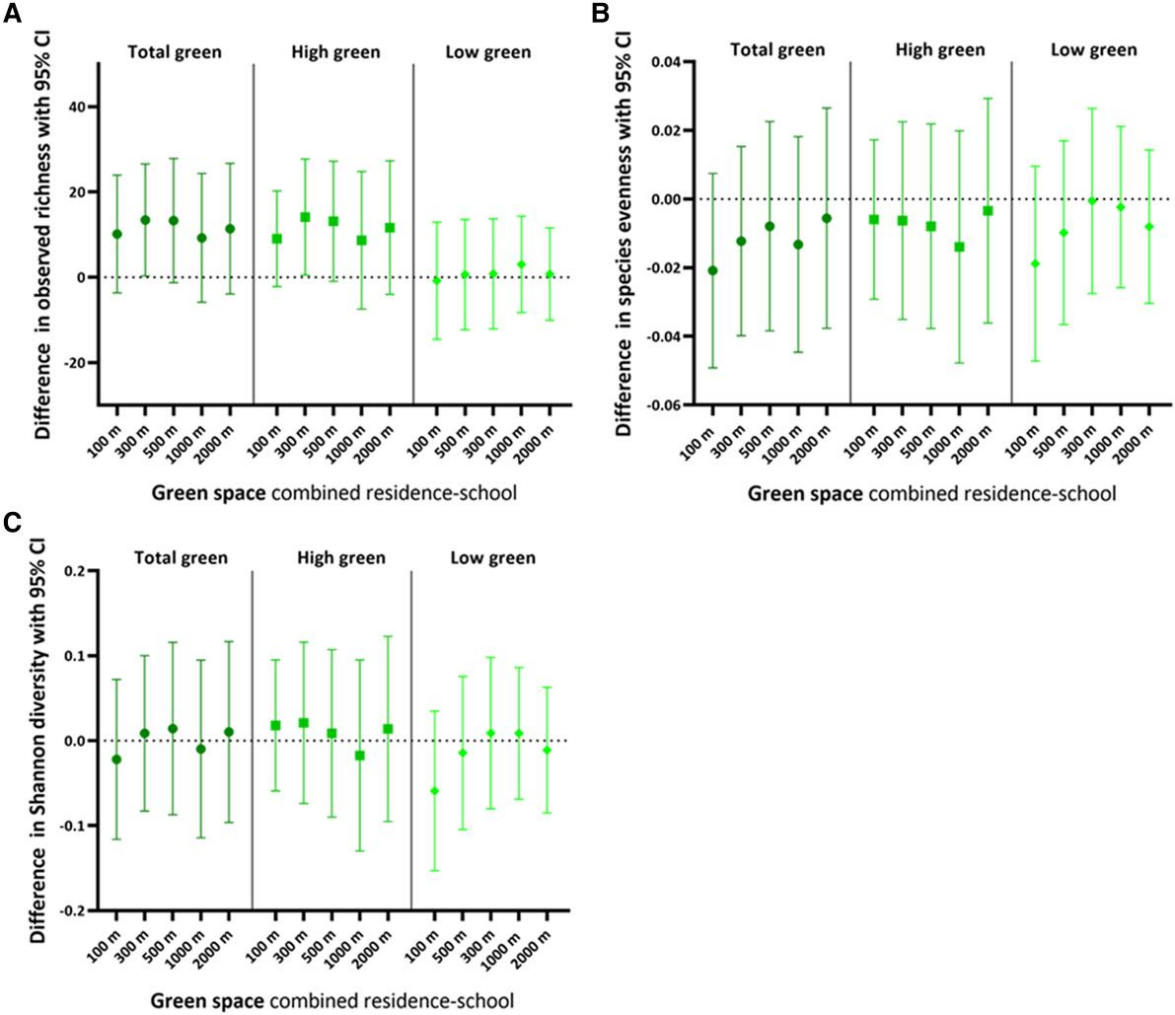
Association between green space exposure (combined residence-school) in different radii and skin microbiome alpha diversity indices: A) observed richness, B) species evenness, and C) Shannon diversity. Green space was defined as total green, high-growing green (vegetation >3 m), and low-growing green (vegetation <3 m). Multiple linear regression models were adjusted for the child's sex, age, frequency of soap use, maternal education, season of skin swab collection, sequencing batch, and storage duration of the skin swab. The estimates represent the difference in alpha diversity with a 95% CI per IQR increase in green space exposure in the respective radius. See Table S2 for corresponding numeric data. *n* = 380.

We conducted multiple linear regression analyses to evaluate the association between exposure to green space and the skin microbiome's alpha diversity. We found that total green and high-growing green (combined residence and school) in multiple radii (from 100 to 500 m) were statistically significantly positively associated with observed richness (Fig. 2A). For instance, an IQR (17%) increase in total green within 300 m was associated with a 14.35 (95% CI: 1.26 to 27.44; *P* = 0.03) higher observed richness. With regard to high-growing green, an IQR (17%) increase in high-growing green within 300 m was associated with a 15.31 (95% CI: 1.73 to 28.90; *P* = 0.03) higher observed richness, and an IQR (18%) increase in high-growing green within 500 m with a 14.27 (95% CI: 0.18 to 28.35; *P* = 0.05) higher observed richness. In addition, total green within 500 m and high-growing green within 100 m were borderline significantly associated with observed richness (total green 500 m: 14.16 [*P* = 0.06] and high-growing green 100 m: 10.06 [*P* = 0.08]). No significant associations were observed between low-growing green and bacterial richness. Likewise, no statistically significant associations were observed between green space exposure and species evenness or Shannon diversity (Fig. 2B and C). In sensitivity analyses (Table S1), we showed that additionally adjusting for furry pets, ethnicity, short-term air pollution exposure (prior week PM_2.5_), long-term air pollution exposure (prior year PM_2.5_), passive smoke exposure, indoor mold, parity, skin disorders, previous month or year antibiotic use, and time spent outdoors did not importantly change the effect estimates. Similarly, findings remained the same when only green space exposure at the residential address was analyzed or when only children who had lived for at least 1 year at the current residential address (*n* = 366) or only children who were born via a natural delivery (*n* = 368) were included in the analysis.

**Fig. 3.**
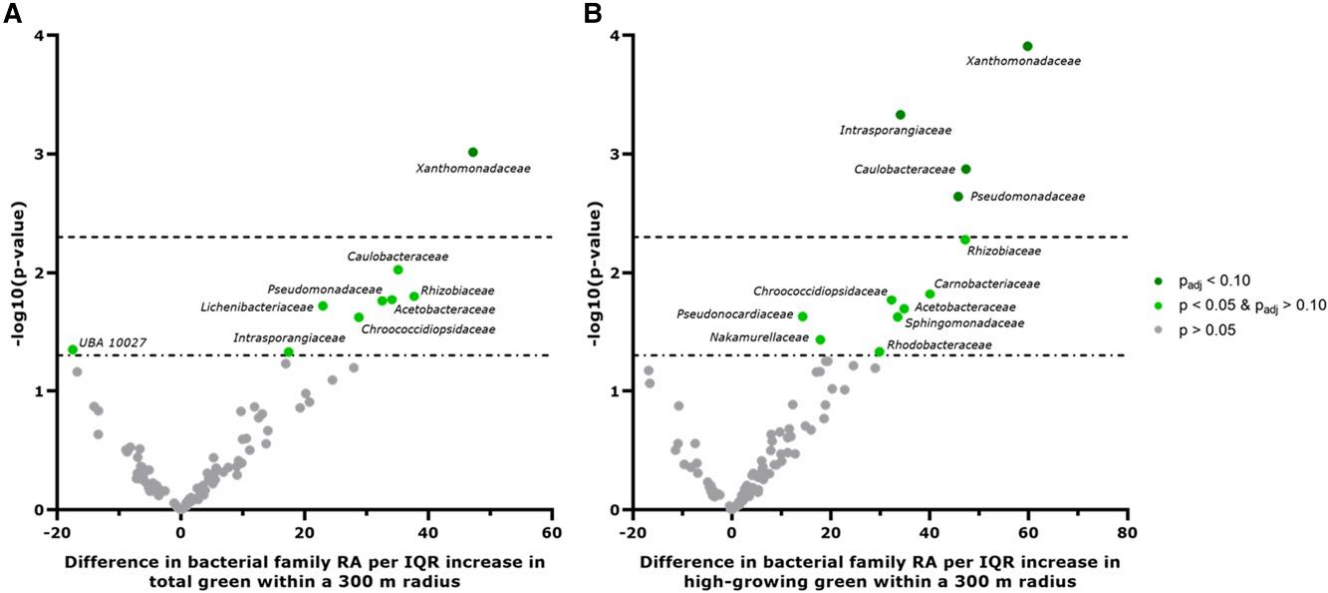
Volcano plot of the associations between A) total green and B) high-growing green exposure and the relative abundance at the bacterial family level. Green space was defined as total green and high-growing green (vegetation >3 m) within 300 m. Multiple linear regression models were adjusted for the child's sex, age, frequency of soap use, maternal education, season of skin swab collection, sequencing batch, and storage duration of the skin swab. Results are expressed as the difference in relative abundance (%) per IQR increase in green space. Statistically significant families (*P* ≤ 0.05) are indicated in light green. Families that remained statistically significant after FDR adjustment (q-value ≤ 0.10) are shown in dark green.

We examined whether our findings were season specific by running multiple linear regression models, including an interaction term between green space and season. As some *P*-values for the interaction terms were borderline statistically significant (Table 3), we stratified the analyses between green space exposure and bacterial alpha diversity indices based on season. Compared with the entire study population, associations between total green and high-growing green were stronger with bacterial richness when skin swabs were collected in the warm season. Furthermore, all the associations for the warm season remained (borderline) statistically significant. For instance, an IQR (19%) increase in high-growing green within 300 m was associated with a 24.30 (95% CI: 2.89 to 45.68; *P* = 0.03) higher observed richness when analyzing the microbiome data from the swabs collected in the warm season. Besides the statistically significant associations found for the entire study population, we found that high-growing green within 100 m was statistically significantly associated with a 14.62 (95% CI: 0.21 to 29.06; *P* = 0.05) higher observed richness when swabs were collected in the warm season. We found no statistically significant associations for the cold season (Table 3). In addition, no associations were found between green space and species evenness or Shannon diversity of the skin swabs collected in the warm or cold season.

**Table 3.** Associations between green space exposure (combined residence and school) in different radii and skin microbiome alpha diversity stratified by season of skin swab collection and *P*-values of the interaction term between green space and season of skin swab collection.

	Observed richness			Species evenness			Shannon diversity		
	Warm	Cold	*P*-value for interaction	Warm	Cold	*P*-value for interaction	Warm	Cold	*P*-value for interaction
	Estimate (95% CI)			Estimate (95% CI)			Estimate (95% CI)		
*Total green*									
100 m	16.74 (−2.12 to 35.61)	0.04 (−20.67 to 20.76)	0.21	−0.01 (−0.04 to 0.02)	−0.03 (−0.09 to 0.03)	0.54	0.01 (−0.11 to 0.13)	−0.08 (−0.13 to 0.20)	0.31
300 m	19.36 (−0.37 to 39.09)	3.72 (−16.05 to 23.49)	0.27	−0.01 (−0.04 to 0.02)	−0.01 (−0.07 to 0.04)	0.77	0.03 (−0.09 to 0.16)	−0.02 (−0.18 to 0.14)	0.41
500 m	22.22 (−0.72 to 45.15)	−1.18 (−20.11 to 17.75)	0.13	−0.01 (−0.05 to 0.03)	−0.001 (−0.05 to 0.05)	0.91	0.03 (−0.12 to 0.17)	−0.01 (−0.16 to 0.14)	0.54
1,000 m	15.01 (−6.65 to 36.67)	−3.78 (−25.24 to 17.67)	0.18	−0.02 (−0.06 to 0.02)	−0.002 (−0.06 to 0.06)	0.66	−0.01 (−0.15 to 0.13)	−0.02 (−0.19 to 0.15)	0.77
2,000m	17.96 (−5.77 to 4.68)	16.20 (−5.20 to 37.59)	0.25	−0.02 (−0.06 to 0.02)	0.02 (−0.04 to 0.07)	0.31	−0.01 (−0.16 to 0.14)	0.04 (−0.13 to 0.20)	0.80
*High-growing green*									
100 m	14.62 (0.21 to 29.06)	−3.01 (−19.45 to 13.43)	0.06	0.01 (−0.02 to 0.03)	−0.02 (−0.07 to 0.02)	0.22	0.06 (−0.03 to 0.15	−0.07 (−0.20 to 0.06)	0.06
300 m	24.30 (2.89 to 45.68)	0.55 (−16.19 to 17.30)	0.08	0.01 (−0.03 to 0.04)	−0.02 (−0.06 to 0.03)	0.36	0.07 (−0.06 to 0.21)	−0.05 (−0.18 to 0.09)	0.15
500 m	21.18 (−1.92 to 44.29)	1.90 (−14.22 to 18.02)	0.19	−0.01 (−0.04 to 0.03)	−0.01 (−0.05 to 0.03)	0.78	0.02 (−0.12 to 0.17)	−0.02 (−0.15 to 0.10)	0.48
1,000 m	13.96 (−11.25 to 39.17)	−0.61 (21.81 to 20.58)	0.32	−0.02 (−0.07 to 0.02)	0.001 (−0.06 to 0.06)	0.53	−0.04 (−0.20 to 0.12)	−0.01 (−0.17 to 0.16)	0.96
2,000m	9.34 (−4.30 to 42.99)	17.85 (−3.97 to 39.67)	0.18	−0.01 (−0.05 to 0.03)	0.01 (−0.04 to 0.07)	0.45	0.004 (−0.16 to 0.14)	0.02 (−0.14 to 0.19)	0.99
*Low-growing green*									
100 m	−2.47 (−22.78 to 17.84)	4.15 (−13.91 to 22.22)	0.40	−0.03 (−0.06 to 0.05)	−0.004 (−0.05 to 0.04)	0.47	−0.10 (−0.23 to 0.03)	0.003 (−0.14 to 0.14)	0.27
300 m	−1.32 (−20.89 to 18.24)	3.82 (−13.32 to 20.96)	0.55	−0.02 (−0.06 to 0.01)	0.01 (−0.03 to 0.06)	0.30	−0.04 (−0.17 to 0.08)	0.04 (−0.20 to 0.18)	0.39
500 m	4.48 (−14.14 to 23.10)	−5.74 (−24.60 to 13.12)	0.50	−0.01 (−0.04 to 0.02)	0.02 (−0.03 to 0.07)	0.46	0.01 (−0.11 to 0.13)	0.03 (−0.12 to 0.18)	0.94
1,000 m	7.42 (−9.06 to 23.89)	−5.36 (−21.05 to 10.33)	0.29	−0.0003 (−0.03 to 0.03)	−0.004 (−0.05 to 0.04)	0.80	0.03 (−0.07 to 0.14)	−0.02 (−0.15 to 0.10)	0.46
2000m	−2.94 (−19.58 to 13.70)	−2.48 (−16.51 to 11.55)	0.57	−0.02 (−0.05 to 0.04)	0.01 (−0.02 to 0.05)	0.14	−0.05 (−0.16 to 0.05)	0.04 (−0.06 to 0.15)	0.23

The table shows the difference in observed richness, species evenness, and Shannon diversity with 95% CI per IQR increase in green space exposure. Green space was defined as total green, high-growing green (vegetation >3 m), and low-growing green (vegetation <3 m). Multiple linear regression models were adjusted for the child's sex, age, frequency of soap use, maternal education, sequencing batch, and storage duration of the skin swab. Seasons were defined based on astronomical seasons. *n* warm = 214. *n* cold = 166.

### Associations between green space exposure and bacterial relative abundances

Raw family and species counts were used as input to the ANCOM-BC R package to examine the association between total green or high-growing green within 300 m and the relative abundance at family and species levels. We have chosen to focus on green space within the 300 m radius, as this radius showed the strongest associations with bacterial richness. Nine families were associated with total green (Fig. 3A), and 12 families with high-growing green within 300 m (Fig. 3B). After multiple testing corrections through FDR, one and four bacterial families remained statistically significantly associated with total green and high-growing green within 300 m, respectively. The bacterial family Xanthomonadaceae was associated with both green-space indices (total green per IQR increment: 47.26%, *q* = 0.09; high-growing green per IQR increment: 59.82%, *q* = 0.01). Three other bacterial families were only associated with high-growing green: Intrasporangiaceae (34.10%, *q* = 0.02), Caulobacteraceae (47.33%, *q* = 0.04), and Pseudomonadaceae (45.75%, *q* = 0.05). No other associations withstood multiple testing corrections. With regard to the species level, 15 and 19 species were associated with total green (Fig. S2A) and high-growing green within 300 m (Fig. S2B), respectively. However, none of the associations remained statistically significant after FDR correction.

## Discussion

To our knowledge, this is the first study assessing the association between early-life exposure to green space exposure and the skin microbiome of children. The key finding of our research was that total green and high-growing green (combined residence and school) in multiple radii (from 100 to 500 m) were positively associated with observed richness. Various sensitivity analyses supported our main findings. The associations were stronger and only statistically significant when skin swabs were collected in the warm season. After multiple testing corrections through FDR, the relative abundance of the bacterial families Xanthomonadaceae, Intrasporangiaceae, Pseudomonadaceae, and Caulobacteraceae was statistically significantly positively associated with total and/or high-growing green within 300 m. These findings indicate that early-life green space exposure may influence skin health, as a decreased skin alpha diversity has been associated with psoriatic lesions, atopic dermatitis, and acne (1, 3–5). In addition, previous studies have shown an intercorrelation between environmental biodiversity, human microbiota, and the risk of allergy development, as atopic individuals had lower biodiversity in the surroundings of their homes and lower diversity of Gammaproteobacteria on their skin (24). Our findings align with existing literature. Pearson et al. (25), for instance, examined the association between the skin microbiome (swabs collected from ears, eyes, nose, and rectum) of 48 deceased adults and neighborhood green remediation (e.g. tree planting, presence of a maintained park, community garden, or urban farm) in Detroit, in the United States. They found positive correlations between microbial evenness and diversity and green remediation. In addition, a study of 188 Finnish teenagers found positive associations between green space exposure (i.e. forest and agricultural land) within 3 km of the residence and the abundance of various skin microbes within the phylum Proteobacteria (24), which is in line with our findings as Xanthomonadaceae, Pseudomonadaceae, and Caulobacteraceae also belong to this phylum.

Exposure to an increased number of microbes during early life, resulting in increased microbial richness on the skin, may impact health in later life, as implicated by the Old Friends Hypothesis (26). According to this hypothesis, increased exposure to microorganisms during the development of the immune system (i.e. during childhood) decreases allergen susceptibility (13) and lowers the prevalence of chronic inflammatory disorders, including allergies and autoimmune disorders (26). This is because certain bacterial strains, e.g. *Corynebacterium* and *Micrococcus*, produce short-chain fatty acids such as butyrate, which have immunoregulatory properties by enhancing the production of regulatory T cells in the skin (26). The Old Friends Hypothesis was supported by a 28-day intervention trial in which urban environmental biodiversity was manipulated to assess effects on the skin microbiome and immunoregulation in children. The intervention diversified skin gammaproteobacterial communities. These higher levels of gammaproteobacterial communities were in turn associated with an increase in immunoregulatory pathways (i.e. higher plasma TGF-B1 levels and more regulatory T cells) (27).

In our study, the associations between green space exposure and skin microbiome alpha diversity were driven by high-growing green but not by low-growing green. We postulate that this difference is due to the difference in the microbial community between trees and grasses/other small plants. Trees have a more complex structure (e.g. roots, bark, and leaves), providing more diverse habitats for microorganisms (28). Thus, it could be that the microbial diversity of trees is higher compared with that of grasses/other small plants. When analyses were stratified based on the season of skin swab collection, positive associations between green space exposure and skin microbiome alpha diversity were only statistically significant in the warm season, while no statistically significant associations were found in the cold season. Furthermore, the associations were stronger compared with the results of the entire study population. A plausible reason for this might be that children spent more time outdoors in the warm season compared with the cold season (7 versus 5 times/week in our study) (29). In addition, the microbial diversity of plants changes with the season, with a statistically significant decrease in colder seasons (30), implying that a lower variety of bacteria might be transferred to humans in colder seasons.

It should also be noted that the number of skin swabs in the cold season was lower than in the warm season (*n* = 166 versus *n* = 214), possibly resulting in a lack of power to detect significant associations. While positive trends were seen for the associations between green space in all radii and skin bacterial richness, associations were only statistically significant in smaller radii (100 to 500 m). This suggests that a direct proximity to green space has a more significant impact than more distant green space.

Four bacterial families were positively associated with total green and/or high-growing green within 300 m. The bacterial family Xanthomonadaceae was associated with both green-space indices. It produces protease enzymes to hydrolyze keratin (31). Keratin is an important skin compound as it forms intermediate filaments in the epidermis (32) to maintain skin integrity by creating a fibrous network (32). Additionally, due to its water impermeability, keratin avoids skin dehydration (33). Patients with skin disorders such as psoriasis often have low skin levels of Xanthomonadaceae family members (e.g. *Xanthomonas*), leading to high keratin levels and epidermal hyperproliferation (34). In a study in patients with psoriasis vulgaris (34), a balneotherapy treatment with *Xanthomonas*-enriched La Roche-Posay thermal spring water was performed to investigate potential clinical improvement. After the balneotherapy, the relative abundance of *Xanthomonas* increased, accompanied by a decrease in the Psoriasis Area and Severity index score on both affected and unaffected skin areas. A similar increase in *Xanthomonas* and improved skin health was observed in atopic dermatitis patients treated for 28 days with an emollient enriched with nonpathogenic bacteria, such as *Xanthomonas* (35). The second bacterial family positively associated with high-growing green was Intrasporangiaceae. Although the biological function of Intrasporangiaceae on human skin remains unstudied, its relative abundance is higher on the skin of healthy individuals compared with patients with moderate or severe atopic dermatitis (36). A study in Brazil examined the household and skin microbiome along an urbanization gradient in the Amazon rainforest and sampled 10 houses and their inhabitants at five locations. They reported that the relative abundance of, among others, Intrasporangiaceae decreased inside the house and on human skin with an increase in urbanization (37). This is in line with the positive association between green space exposure and the relative abundance of Intrasporangiaceae found in our study. The third bacterial family associated with high-growing green, Pseudomonadaceae, is found in water sources (e.g. hot tubs and swimming spools), and its relative abundance is negatively associated with skin transepithelial water loss and sebum levels in a study in Chinese women (38), reflecting its role in proper skin barrier functioning. Pseudomonadaceae contains opportunistic pathogens, such as *Pseudomonas aeruginosa* (39). An infection with this bacterial strain can cause effects ranging from local skin symptoms (e.g. hot tub folliculitis and hot hand–foot infection) to life-threatening bacteremia (40, 41). Nevertheless, severe infections are rarely seen in healthy individuals but rather in immunocompromised patients, such as patients with cancer, burn injuries, or nonhealing diabetic wounds (39). The fourth bacterial family positively associated with high-growing green exposure is Caulobacteraceae, inhabiting, among others, soil, plants, and seawater (42). A study in adults with chronic plaque psoriasis reported a lower relative abundance of Caulobacteraceae on leg lesions than on nonlesioned skin (43). Even though the biological function of Caulobacteraceae on human skin is not investigated, it has well-characterized alkaline phosphatase activity (42, 44). Alkaline phosphate is essential for skin health through its involvement in wound healing, as shown in a study in Wistar rats (45). In this study, skin wounds were generated and alkaline phosphate levels were measured in the healing tissue at different time points. The researchers noticed a significant increase in alkaline phosphate levels initially, which decreased over time as healing progressed (45).

This study has some limitations. We investigated the skin microbiome by collecting skin swabs to provide a standardized, user-friendly, and noninvasive approach to examine the superficial microbiome composition (46). We acknowledge that microorganisms inhabiting deeper skin layers, often important for dermal skin diseases, could not be captured and investigated this way (46, 47). Yet, collecting skin biopsies is invasive (47) and will probably result in a low participation rate. Furthermore, multiple linear regression models were corrected for the frequency of soap use but not specifically for the use of soap on the face. In addition, we had no information on the time since the last bathing/washing; therefore, we could not include that as a variable in the sensitivity analyses. Our analyses combined green space at the residential and school address. We acknowledge that children spend less time at the school address in the warm season. Therefore, we also ran the stratified analyses only considering residential green space and obtained results mainly similar to the combined exposure analyses (data not shown). Our study also has various strengths. First, green space exposure was combined at the residential and school addresses because the school environment also influences children's behavior and health (48, 49). Second, we were able to adjust for many potential confounders and performed sensitivity analyses to support our findings further. Furthermore, due to the large sample size, we were able to stratify based on the season of skin swab collection. Last, the skin microbiome profile was assessed with high reliability using 16S Pacbio HiFi, which sequences the full-length 16S rRNA gene. This results in long, highly accurate, single-molecule consensus reads, of which up to 90% can be functionally annotated at the species level (50). Large-scale studies should confirm our findings. In addition, future research should investigate the mechanisms through which green spaces can transfer bacteria to humans. Therefore, participants should be exposed to specific natural elements (e.g. pollen, leaves, and soil) in controlled settings to see how they can impact the skin microbiome composition.

## Conclusion

We assessed the association between early-life exposure to green space and the microbiome alpha diversity and the relative abundance of bacterial families and species of children's skins. We found that total green and high-growing green in multiple radii around the residence and school were positively associated with bacterial richness. When stratified based on skin microbiome sampling season, the observed associations were stronger and only statistically significant when skin swabs were collected in spring and summer. After correction for multiple testing, the relative abundance of the bacterial families Xanthomonadaceae, Intrasporangiaceae, Pseudomonadaceae, and Caulobacteraceae was significantly positively associated with total and/or high-growing green within 300 m. None of the associations with bacterial species withstood correction for multiple testing. Further research is needed to determine whether the observed positive association between green space and skin bacterial richness has implications for human (skin) health, as this would highlight the importance of ensuring adequate green space in residential and school environments further.

## Supplementary Material

pgaf115_Supplementary_Data

## Data Availability

The full personal data come from the ENVIR*ON*AGE Birth Cohort, which is proprietary and cannot be deposited in a public repository. Data from the ENVIR*ON*AGE birth cohort can be requested from tim.nawrot@uhasselt.be.
