# Supplementary material for: Exposure to green space is associated with higher skin microbiota species richness in children
Source: PNAS Nexus. 2025 May 19;4(5):pgaf115. doi: 10.1093/pnasnexus/pgaf115 (PMC12087450; doi:10.1093/pnasnexus/pgaf115)
Supplement: pgaf115_Supplementary_Data [file pgaf115_supplementary_data.docx]

***Supplementary Material***

**Exposure to Green Space is Associated with Higher Skin Microbiota Species Richness in Children**

Thessa Van Pee^1^, Hanne Croons^1^, Esmée Bijnens^1,2^, Doris Vandeputte^3^, Eleni Renaers^1^, Hanne Sleurs^1^, Lore Verheyen^1^, Nick Giesberts^1^, Maartje Vangeneugden^1^, Leen Rasking,^1^ Michelle Plusquin^1^, Janneke Hogervorst^1^, Tim S. Nawrot^1,4^

^1^Centre for Environmental Sciences, Hasselt University, Agoralaan Building D, 3590, Diepenbeek, Belgium

^2^Open Universiteit, Valkenburgerweg 177, 6419, Heerlen, Nederland

^3^Lab of Microbiology LM-UGent, Department of Biochemistry and Microbiology (WE10), Ghent University, K.L. Ledeganckstraat 35, 9000 Ghent

^4^Department of Public Health and Primary Care, Leuven University, Herestraat 49-box 706, 3000, Leuven, Belgium

*To whom correspondence should be addressed: Tim Nawrot, Tel: +3211268382; Email: tim.nawrot@uhasselt.be, Agoralaan building D, 3590, Diepenbeek, Belgium

**Declaration of conflicts of interest**: all authors declare no conflict of interest.

**Table S1.** Sensitivity analyses of associations between green space exposure and skin microbiome observed richness after additional correction for furry pets, ethnicity, short-term air pollution exposure (prior week PM_2.5_), long-term air pollution exposure (prior year PM_2.5_), skin disorders, time spent outdoors, passive smoke exposure, indoor molt, or parity, or only considering green space exposure at the residence, only including children who lived more than one year at the current residence, or only including children born via a natural delivery.

| **Additionally corrected for/ exclusion of** | **Green space** | **Observed richness** | |
| --- | --- | --- | --- |
|  |  | Change | 95% CI |
| **Furry pets** | Total green – 300 m | 16.52 | 1.61 to 31.43 |
|  | Total green – 500 m | 13.30 | -1.19 to 27.80 |
|  | High green – 100 m | 9.07 | -2.03 to 20.20 |
|  | High green – 300 m | 18.07 | 2.59 to 33.54 |
|  | High green – 500 m | 16.18 | 0.74 to 31.62 |
| **Ethnicity** | Total green – 300 m | 14.33 | 1.22 to 27.44 |
|  | Total green – 500 m | 13.24 | -1.33 to 27.80 |
|  | High green – 100 m | 8.94 | -2.23 to 20.12 |
|  | High green – 300 m | 15.27 | 1.64 to 28.91 |
|  | High green – 500 m | 14.23 | 0.09 to 28.36 |
| **Modeled PM_2.5_ exposure during the prior week** | Total green – 300 m | 14.22 | 0.96 to 27.32 |
|  | Total green – 500 m | 13.39 | -1.17 to 27.95 |
|  | High green – 100 m | 8.83 | -2.34 to 19.99 |
|  | High green – 300 m | 15.36 | 1.77 to 28.95 |
|  | High green – 500 m | 14.48 | 0.39 to 28.58 |
| **Modeled PM_2.5_ exposure during the prior year** | Total green – 300 m | 13.96 | 0.71 to 27.19 |
|  | Total green – 500 m | 12.87 | -1.92 to 27.66 |
|  | High green – 100 m | 8.77 | -2.41 to 25.21 |
|  | High green – 300 m | 14.95 | 1.28 to 28.64 |
|  | High green – 500 m | 13.86 | -0.34 to 28.06 |
| **Skin disorders** | Total green – 300 m | 14.85 | 1.72 to 27.98 |
|  | Total green – 500 m | 13.68 | -0.96 to 28.32 |
|  | High green – 100 m | 9.24 | -1.94 to 20.42 |
|  | High green – 300 m | 15.70 | 2.09 to 29.31 |
|  | High green – 500 m | 14.81 | 0.68 to 28.94 |
| **Previous month antibiotic use** | Total green – 300 m | 16.41 | 0.99 to 31.83 |
|  | Total green – 500 m | 13.28 | -1.29 to 27.86 |
|  | High green – 100 m | 9.04 | -2.16 to 20.24 |
|  | High green – 300 m | 15.11 | -0.88 to 31.10 |
|  | High green – 500 m | 14.45 | -1.42 to 30.32 |
| **Previous year antibiotic use** | Total green – 300 m | 13.47 | 0.34 to 26.60 |
|  | Total green – 500 m | 13.27 | -1.29 to 27.83 |
|  | High green – 100 m | 8.97 | -2.19 to 20.12 |
|  | High green – 300 m | 13.28 | 0.34 to 26.22 |
|  | High green – 500 m | 12.02 | -1.17 to 25.21 |
| **Time spent outdoors** | Total green – 300 m | 16.41 | 0.99 to 31.83 |
|  | Total green – 500 m | 13.28 | -0.77 to 29.04 |
|  | High green – 100 m | 8.25 | -3.28 to 19.79 |
|  | High green – 300 m | 15.11 | -0.88 to 31.10 |
|  | High green – 500 m | 14.45 | -1.42 to 30.32 |
| **Passive smoke exposure*** | Total green – 300 m | 16.48 | 3.26 to 29.71 |
|  | Total green – 500 m | 14.84 | 0.35 to 29.32 |
|  | High green – 100 m | 10.07 | -1.07 to 21.20 |
|  | High green – 300 m | 15.45 | 1.87 to 29.04 |
|  | High green – 500 m | 14.49 | 0.45 to 28.52 |
| **Indoor mold*** | Total green – 300 m | 15.19 | 2.07 to 28.30 |
|  | Total green – 500 m | 13.72 | -0.61 to 28.04 |
|  | High green – 100 m | 8.48 | -2.53 to 19.49 |
|  | High green – 300 m | 14.79 | 1.36 to 28.05 |
|  | High green – 500 m | 13.48 | -0.40 to 27.36 |
| **Parity** | Total green – 300 m | 14.09 | 0.93 to 27.24 |
|  | Total green – 500 m | 13.20 | -1.35 to 27.76 |
|  | High green – 100 m | 8.36 | -2.86 to 19.58 |
|  | High green – 300 m | 14.90 | 1.17 to 28.54 |
|  | High green – 500 m | 14.16 | 0.01 to 28.13 |
| **Residential green space only** | Total green – 300 m | 5.56 | -1.61 to 12.73 |
|  | Total green – 500 m | 8.82 | -0.99 to 18.62 |
|  | High green – 100 m | 7.93 | -0.69 to 16.55 |
|  | High green – 300 m | 15.37 | 2.10 to 28.33 |
|  | High green – 500 m | 14.55 | 0.77 to 28.33 |
| **Only children who have lived >1 year at the current address (n = 366)** | Total green – 300 m | 16.64 | 1.37 to 31.91 |
|  | Total green – 500 m | 14.44 | -0.39 to 29.28 |
|  | High green – 100 m | 9.27 | -2.00 to 20.13 |
|  | High green – 300 m | 16.05 | 0.12 to 1.98 |
|  | High green – 500 m | 14.66 | -1.18 to 30.50 |
| **Only children born via a natural delivery (n = 368)** | Total green – 300 m | 12.74 | -0.51 to 25.98 |
|  | Total green – 500 m | 12.21 | -0.83 to 22.69 |
|  | High green – 100 m | 10.35 | -1.03 to 17.22 |
|  | High green – 300 m | 14.28 | 0.35 to 28.21 |
|  | High green – 500 m | 14.89 | 0.61 to 29.18 |

The table shows the difference in observed richness with 95% CI per IQR increase in green space. Green space was defined as total green and high-growing green (vegetation higher than 3 m). Multiple linear regression models were adjusted for the child’s sex, age, frequency of soap use, maternal education, season of skin swab collection, sequencing batch, and storage duration of the skin swab. CI: confidence interval; IQR: interquartile range. n = 380 or as indicated. *Data available for 362 participants.

**Table S2.** Numeric data of the associations between green space (combined residence-school) in different radii and skin microbiome alpha diversity indices.

|  | **Observed richness** | | | **Species evenness** | | | **Shannon diversity** | | |
| --- | --- | --- | --- | --- | --- | --- | --- | --- | --- |
|  | estimate | 95% CI | p-value | estimate | 95% CI | p-value | estimate | 95% CI | p-value |
| **TOTAL GREEN** | | | | | | | | | |
| **100 m** | 10.89 | -2.70 to 24.49 | 0.12 | -0.02 | -0.05 to 0.007 | 0.15 | -0.02 | -0.12 to 0.72 | 0.64 |
| **300 m** | 14.35 | 1.26 to 27.44 | 0.03 | -0.01 | -0.04 to 0.02 | 0.38 | 0.009 | -0.08 to 0.10 | 0.85 |
| **500 m** | 14.16 | -0.36 to 28.67 | 0.06 | -0.008 | -0.04 to 0.02 | 0.61 | 0.02 | -0.09 to 0.12 | 0.78 |
| **1000 m** | 10.24 | -4.82 to 25.29 | 0.18 | -0.01 | -0.05 to 0.02 | 0.41 | -0.01 | -0.11 to 0.10 | 0.86 |
| **3000 m** | 12.16 | -3.20 to 27.51 | 0.12 | -0.006 | -0.04 to 0.03 | 0.73 | 0.01 | -0.10 to 0.12 | 0.85 |
| **HIGH-GROWING GREEN** | | | | | | | | | |
| **100 m** | 10.06 | -1.06 to 21.17 | 0.08 | -0.006 | -0.03 to 0.02 | 0.61 | 0.02 | -0.06 to 0.10 | 0.64 |
| **300 m** | 15.31 | 1.73 to 28.90 | 0.03 | -0.006 | -0.04 to 0.02 | 0.65 | 0.02 | -0.07 to 0.12 | 0.66 |
| **500 m** | 14.27 | 0.18 to 28.35 | 0.05 | -0.008 | -0.04 to 0.02 | 0.60 | 0.009 | -0.09 to 0.10 | 0.86 |
| **1000 m** | 10.14 | -6.01 to 26.29 | 0.22 | -0.01 | -0.05 to 0.02 | 0.42 | -0.02 | -0.13 to 0.10 | 0.76 |
| **3000 m** | 12.45 | -3.25 to 28.15 | 0.12 | -0.003 | -0.04 to 0.03 | 0.84 | 0.01 | -0.10 to 0.12 | 0.80 |
| **LOW-GROWING GREEN** | | | | | | | | | |
| **100 m** | -1.25 | -14.88 to 12.39 | 0.86 | -0.02 | -0.05 to 0.01 | 0.19 | -0.06 | -0.15 to 0.04 | 0.22 |
| **300 m** | 0.50 | -12.33 to 13.33 | 0.94 | -0.01 | -0.04 to 0.02 | 0.47 | -0.02 | -0.10 to 0.08 | 0.74 |
| **500 m** | 0.70 | -12.16 to 13.56 | 0.92 | -0.001 | -0.03 to 0.03 | 0.97 | 0.009 | -0.08 to 0.10 | 0.83 |
| **1000 m** | 2.72 | -8.51 to 13.94 | 0.64 | -0.002 | -0.03 to 0.02 | 0.84 | 0.009 | -0.07 to 0.09 | 0.82 |
| **3000 m** | 0.72 | -10.05 to 11.49 | 0.90 | -0.008 | -0.03 to 0.02 | 0.48 | -0.01 | -0.09 to 0.06 | 0.77 |

The estimates represent the change in alpha diversity with 95% CI per IQR increase in green space in the respective radius. Multiple linear regression models were adjusted for the child’s sex, age, frequency of soap use, maternal education, season of skin swab collection, sequencing batch, and storage duration of the skin swab. n = 380. CI: confidence interval; IQR: interquartile range.


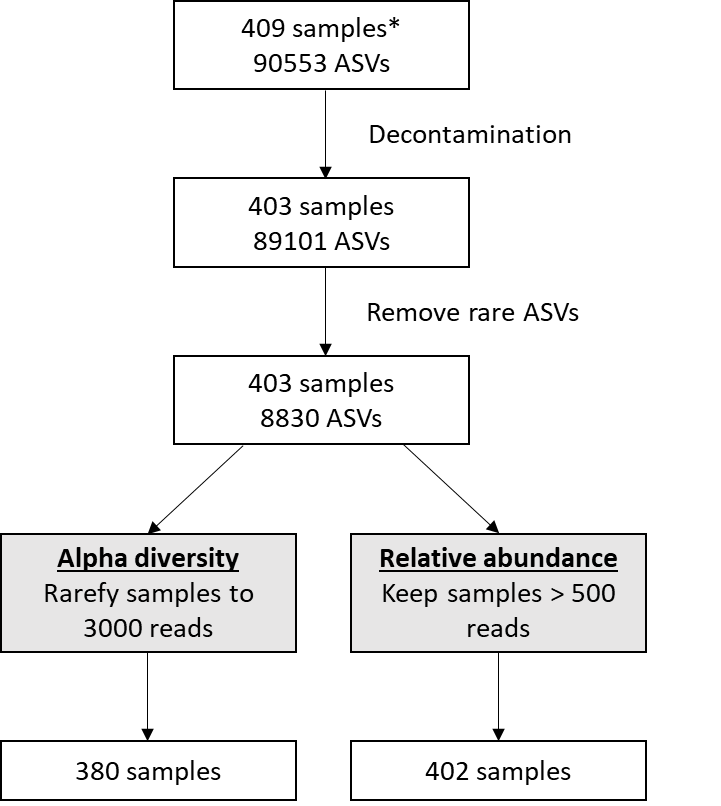


**Figure S1.** Flow chart depicting the flow of samples and amplicon sequence variants (ASVs) to come to the final sample size for the different analyses. *403 biological samples and 6 negative control samples.


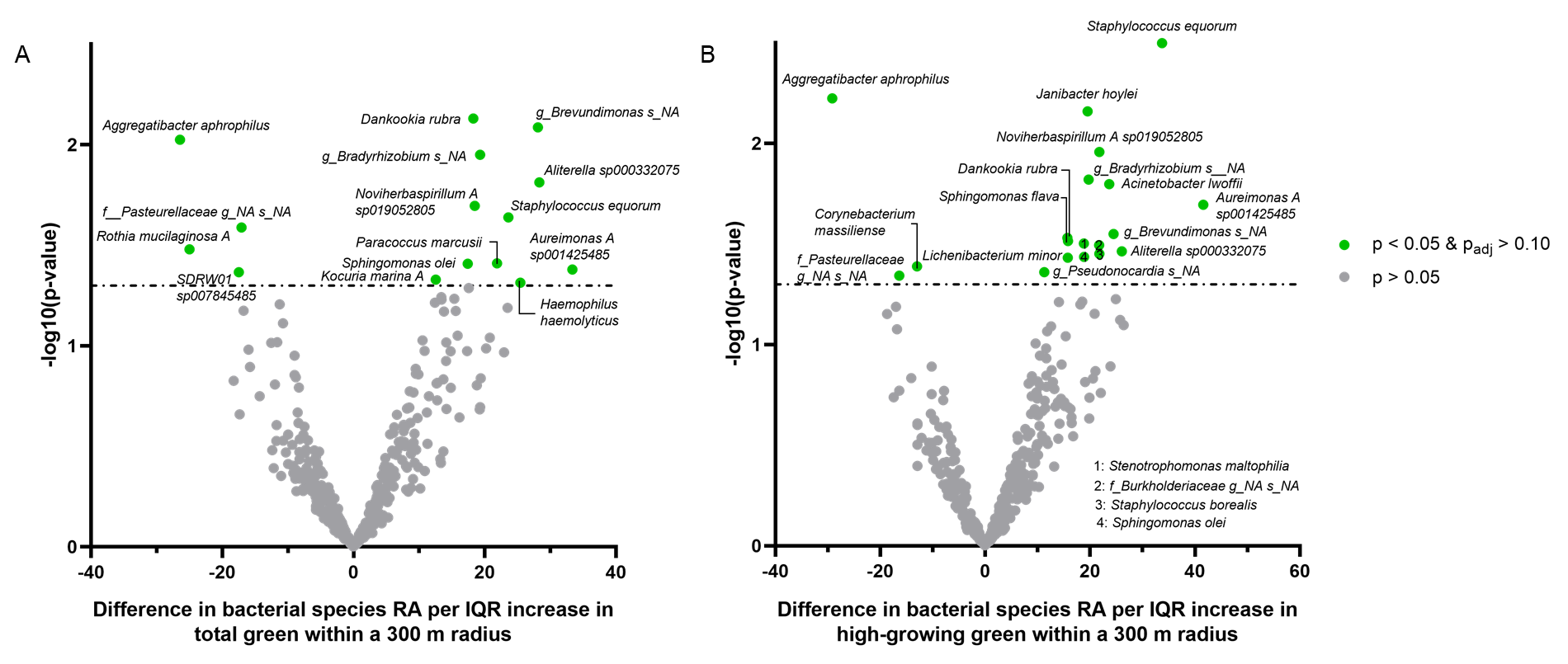


**Figure S2. Volcano plot of the associations between (A) total green and (B) high-growing green and the relative abundance at the bacterial species level.** Green space was defined as total green and high-growing green (vegetation higher than 3 m) within 300 m. Multiple linear regression models were adjusted for the child’s sex, age, frequency of soap use, maternal education, season of skin swab collection, sequencing batch, and storage duration of the skin swab. Results are expressed as the difference in relative abundance (%) per IQR increase in green space. Statistically significant species (p-value ≤ 0.05) are indicated in light green. FDR: false discovery rate; IQR: interquartile range.
